# Prevention of Hypothermia in Surgical Neonates in Operation Theatre: Recommendations of Neonatal Surgery Research Group

**Published:** 2012-01-01

**Authors:** Mumtaz Qureshi

**Affiliations:** Department of Pediatric surgery, National Institute of Child Health Karachi, Pakistan

**Dear Sir,**

Hypothermia in surgical neonates is associated with serious implications and during surgery may lead to the damage to various organs of body and therefore affects the ultimate outcome. Neonatal hypothermia affects the vital organ’s circulation such as cerebral, myocardial and renal and these organs are more sensitive to resultant ischemia [1].

The important factors which lead to hypothermia in operation room are the type and duration of surgery, and operating room temperature as neonate have poorly developed thermoregulatory mechanism and minor changes related with operating room environment, equipments and instruments that used to contact with neonatal body may produce severe hypothermia [1].

In different setups different precautions are taken to prevent the hypothermia during neonatal surgery for example automatic warming system.

- Illumination (consists of two halogen bulbs of 50 watts).
- Warming sources (Two glass rod elements of 750 watts).
- Warming control system (A sensor with a servo mode).
- Alarm system.
- Special Bed for neonate.

Unfortunately in majority of setups proper warming system in neonatal/pediatric surgical units have not been available. This aspect of neonatal surgical care was discussed in the Neonatal Surgery research Group (NSRG) which is a discussion group on face book. Many learned members shared their experiences and advocated recommendations for optimal management and prevention of hypothermia while operation in settings that lack proper thermoregulatory equipments [2]. These recommendations are summarized as under;

1. Infusion of warm intravenous fluids during surgery.
2. Cotton roll/aluminum foils/woolen cap and socks cover over head and limbs.
3. Operating room air conditioner must be off.
4. Use of warm sterilized towels, pyodine solution and surgical instruments or at least they should not be cold.
5. There should not be frequent opening of the doors while operation.
6. Warming pads around baby and warming pad under him may be used.
7. Overhead warmer can be used to control the cold environment especially in winter.

These measures are easy to observe and do not require special instrument/techniques to be employed. Fan heaters or electric heaters must not be used near the neonates as they can directly heat them and may burn the delicate body of neonates. These measures are simple and can avoid hypothermia while operation and thus benefit the outcome of surgical neonates.

## Footnotes

**Source of Support:** Nil

**Conflict of Interest:** None declared

